# Reduction of structural hierarchy translates into variable influence on the performance of boron nitride aerogel

**DOI:** 10.1016/j.isci.2021.102251

**Published:** 2021-03-01

**Authors:** Jingjing Pan, Jingyang Wang

**Affiliations:** 1 Shenyang National Laboratory for Materials Science, Institute of Metal Research, Chinese Academy of Sciences, Shenyang 110016, China; 2 School of Materials Science and Engineering, University of Science and Technology of China, Hefei 230026, China

**Keywords:** Ceramics, Microstructure, Supramolecular Technologies

## Abstract

The rise of ceramic aerogel offers traditional ceramics a new window. Alongside the emerging prospects, what is open to explore includes the elegant design of a ceramic aerogel with tailorable inner organizations, what would occur when complex hierarchy exists in such an already intricate system, and how the properties get influenced as the complexity fades. Borrowing the wisdom from supramolecular world, we exquisitely transform BN aerogel from a complex hierarchy to a flatten microstructure based on solvent-induced morphology switch of its supramolecular precursor gel. Such reduction in structural hierarchy has insignificant effect on the thermal conductivity (∼0.027 W/(m·K)) but shifts the wettability from hydrophobicity to hydrophilicity and occasions nearly 3-fold difference in ion adsorption rate, as exemplified by lead ions. This work may promote the understanding of special hierarchy existing in delicate systems and inspire other attempts to harness the functionality of aerogels by manipulating structural hierarchy.

## Highlights

• Tuning the inner organizations of BN aerogel in light of supramolecular wisdom
• Shifting the wettability from hydrophobicity to hydrophilicity
• Structural hierarchy contributes to much higher adsorption rate for metal ions
• Thermal insulation remains stable despite the change in microstructure features

## Graphical abstract

**Figure:**
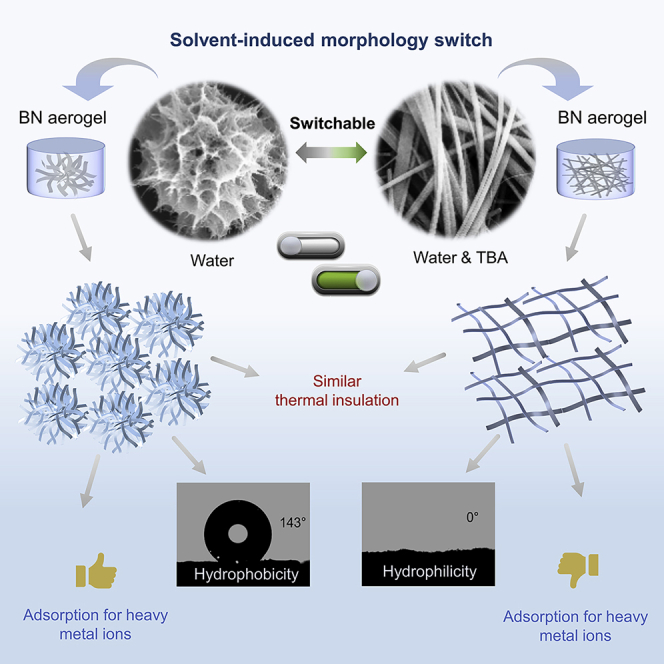

## Introduction

Nature excels in assembling similar building units into various biological systems, in which different levels of organization and complexity translate to versatile functions. As a ubiquitous phenomenon in nature, molecular assembly contributes to the rise of supramolecular chemistry, a realm inspiring the design of intricate and functional architectures in artificial systems (Dumele et al., 2020; Leclercq et al., 2019; Mako et al., 2019; Meneghin et al., 2020). As the “glue” of supramolecular chemistry, a variety of non-covalent interactions (e.g., hydrogen bonds, charge interactions, and coordination) have been utilized for the organization of small components into fascinating systems with various dimensions (Chakrabarty et al., 2011). For instance, metal-organic frameworks, promising in fields like adsorption (Li et al., 2020) and catalysis (Lee et al., 2009), are exactly based on the complexion between metal ions and organic ligands. Other supramolecular architectures, maintained by other non-covalent forces, such as hydrogen bonding (Bui et al., 2020) or pi-pi stacking (Deng et al., 2020), are also primary examples.

Although the arts of non-covalent interactions function well in the supramolecular world, much of this chemistry is limited to relatively mild conditions, as the non-covalent interactions are vulnerable to harsh environments (e.g., high temperature), which limits such superb artistry to certain domains.

In startling contrast, ceramics, dating from the arts of “clay and fire,” are adapted to all kinds of extreme circumstances. Yet, this field has few exquisite tools available—the flexibility in designing ceramics of subtlety is nowhere near matchable to that in constructing diverse supramolecular assemblies, either in biological or in artificial systems. To tap their functionality, ceramics are usually transformed into porous forms. Except for few examples stemming from special techniques (e.g., 3D printing [Eckel et al., 2016]), more commonly seen in many porous ceramics—derived from foaming method (Kim et al., 2019) or freeze casting (Shao et al., 2020), for example—are thick walls and rigid skeletons, which hamper the fulfilling of versatile functions and the unveiling of the new possibilities led by unique structures. Generally, it is prohibitive to explore what would happen when a ceramic exists in a delicate and complex manner.

Recently, the rise of ceramic aerogel has opened up new opportunities (Harley-Trochimczyk et al., 2016; Luo et al., 2019; Si et al., 2018; Wang et al., 2020; Xu et al., 2019; Zhang et al., 2020). Characterized by its nanoscale skeleton, aerogel extends the unique features in nanoscale to macro-world and unlocks the potential of many traditional materials. Existing in aerogel forms, some orthodox ceramics such as boron nitride and silicon carbide, which are well documented to possess intrinsic high thermal conductivity, have been revealed to exhibit super thermal insulation (Wang et al., 2020; Xu et al., 2019). Also, ceramic aerogel shows potential in other fields, such as catalysis (Luo et al., 2019) and sensing (Harley-Trochimczyk et al., 2016). Conceivably, more intriguing properties imparted by the special form of existence will be unveiled alongside the further devotion to this field.

The emerging prospects, meanwhile, lay several aspects open to explore. (1) How can we flexibly design a certain ceramic aerogel with different inner organizations? (2) What would appear if special hierarchy exists in a ceramic aerogel, which is already an intricate system? (3) How the performance will get influenced as such complexity degrades? In the responses to these aspects lay the secret of how we might unleash and harness the functionality of a ceramic aerogel by manipulating structural hierarchy.

Analogous to carbon, boron nitride (BN) is among the most important inorganic material and is able to exist in different nanostructural forms (Arenal and Lopez-Bezanilla, 2015; Golberg et al., 2010; Luo et al., 2017; Yin et al., 2016). Attention paid to BN aerogel could be traced back to the late 20th century when it was first synthesized by a quite arduous approach (Lindquist et al., 1990), whereas the slow advances in fabrication considerably hindered the further exploitation of this material. Recently, on the back of other aerogels (e.g., graphene aerogel, carbon nanotube aerogel and silica aerogel), researchers succeeded in fabricating BN aerogels via template deposition (Rousseas et al., 2013; Song et al., 2015; Thang et al., 2015; Xu et al., 2019; Xue et al., 2017). Meanwhile, there were also attempts to fabricate BN nanosheet aerogels by assembling 2D BN nanosheets (Lei et al., 2015; Zeng et al., 2015) to 3D architectures or to prepare BN nanoribbon aerogels derived from precursors (Li et al., 2019; Lin et al., 2017).

Nonetheless, the elegant fabrication of BN aerogels with special hierarchy—and other ceramic aerogels alike—is quite challenging. In response to this situation, we recently developed an elegant scheme for preparing BN aerogels that embody varied superstructures (Pan and Wang, 2020), whereas little was known about what is accompanied with such complex structure and what would occur if the complex hierarchy fades.

In this work, a lesson is borrowed from supramolecular world that solvent could interfere in the non-covalent interactions and therefore influences the assembly pattern of assembled architectures (Nikoloudakis et al., 2018; Xing et al., 2018; Zhang et al., 2017; Zhu and Dordick, 2006). We successfully transform BN aerogels from a complex hierarchy to a flatten pattern by simply adjusting the initial solvent for the formation of its supramolecular precursor gel. Further explorations reveal that the change in structural hierarchy has little influence on apparent thermal conductivity of the resultant aerogels, whereas it affects the affinity of BN aerogels for water. Interestingly, such hierarchy contrast also leads to varied performance for the adsorption removal of heavy metal ions, for example, lead (Ⅱ) ions. It is found that the absorbed metal ions, i.e., Pb^2+^, show a tendency to reside in tiny localized areas existing in BN aerogel derived from flower-like morphology, a unique hierarchy favorable for the trapping of metal ions. By contrast, BN aerogel with flatten structure has much lower adsorption capacity for the same metal ions, which are mainly distributed on individual nanoribbons. Besides Pb^2+^, Cu^2+^ and Cr^3+^ were additionally chosen for adsorption tests, and the results show that the adsorption capacity of BN aerogel with complex hierarchy still outperforms that of the other aerogel with flatten microstructure, proving that the difference in adsorption performance led by hierarchy disparity also applies to other heavy metal ions.

## Results

### Fabrication and characterization of BN aerogels with different level of structural hierarchy

**Figure 1:**
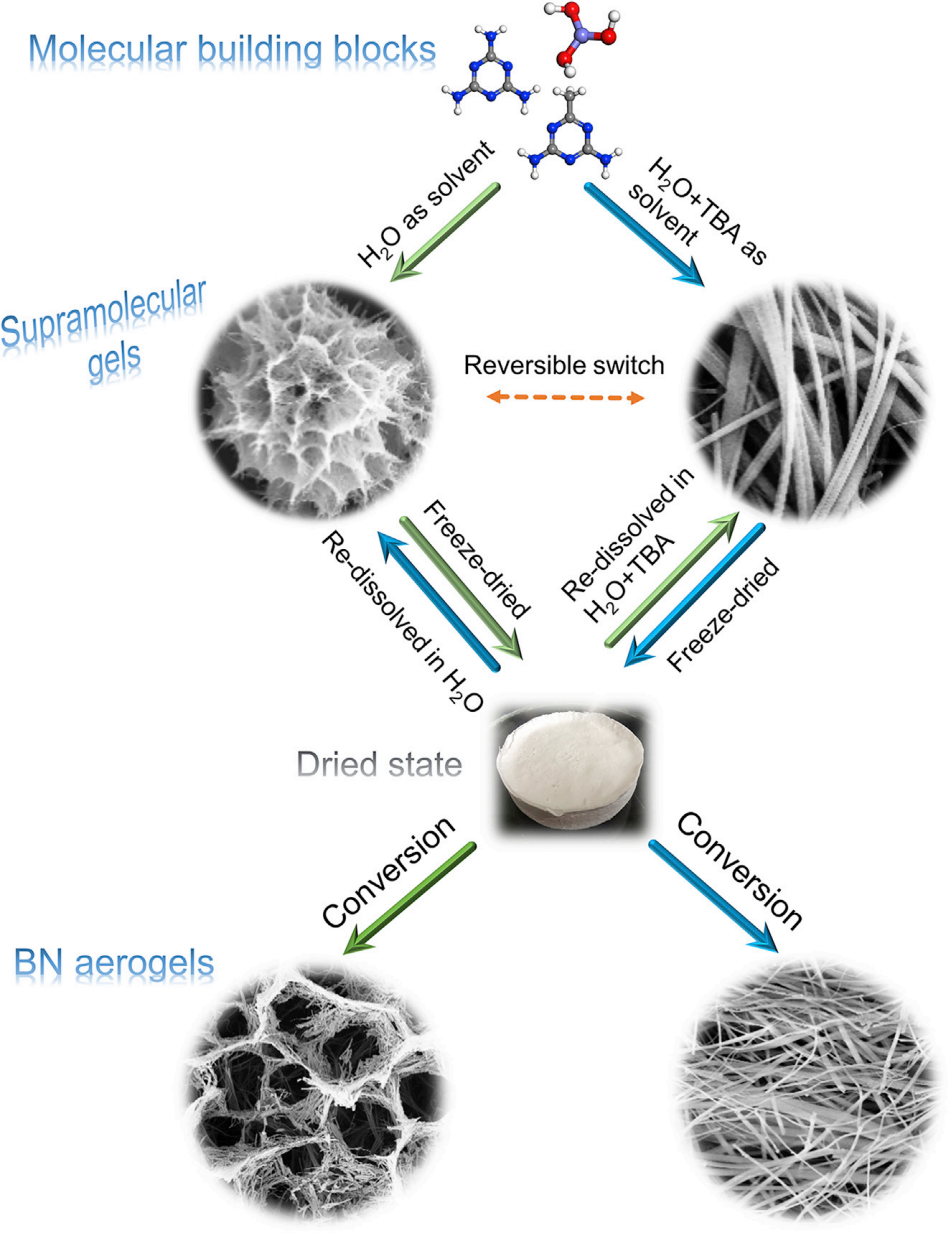
Illustration of solvent-induced morphology switch of supramolecular precursor gels and the resultant BN aerogels with different microstructures Raw molecules are melamine, acetoguanamine, and boric acid.

As depicted in Figure 1, we used pure water and the mixture of water and tert-butyl alcohol (TBA) as different solvents for supramolecular precursor gels, the building molecules for which are melamine (M), acetoguanamine (M∗), and boric acid (B), which could serve as raw materials for the fabrication of BN aerogel with complex inner structure, as described before (Pan and Wang, 2020). These three molecules at a fixed molar ratio (M: M∗: B = 1:1:6) were dissolved in solvents, and wet supramolecular gels formed then as hot solutions were cooled down. After being freeze-dried, the supramolecular gels underwent high-temperature treatment, which initiated their conversion to BN aerogels inheriting the micromorphology of original supramolecular gels (Figure S1).

Clearly, when raw molecules were dissolved in pure water, the as-formed supramolecular gel exhibited a flower-like micromorphology with complex hierarchy, whereas if the mixture of water and TBA was used as solvent (TBA took up 30%), the resultant gel showed a flatten microstructure (Figure 1). What is worthy of mentioning is that the morphology of the above-mentioned supramolecular gels is switchable, i.e., the flatten structure (initial solvent was the mixture of water and TBA) could turn to the flower-like morphology if we re-dissolved the dried supramolecular gels in water, and the flower-like morphology (initial solvent was water), vice versa, could be transformed again into the flatten pattern.

**Figure 2:**
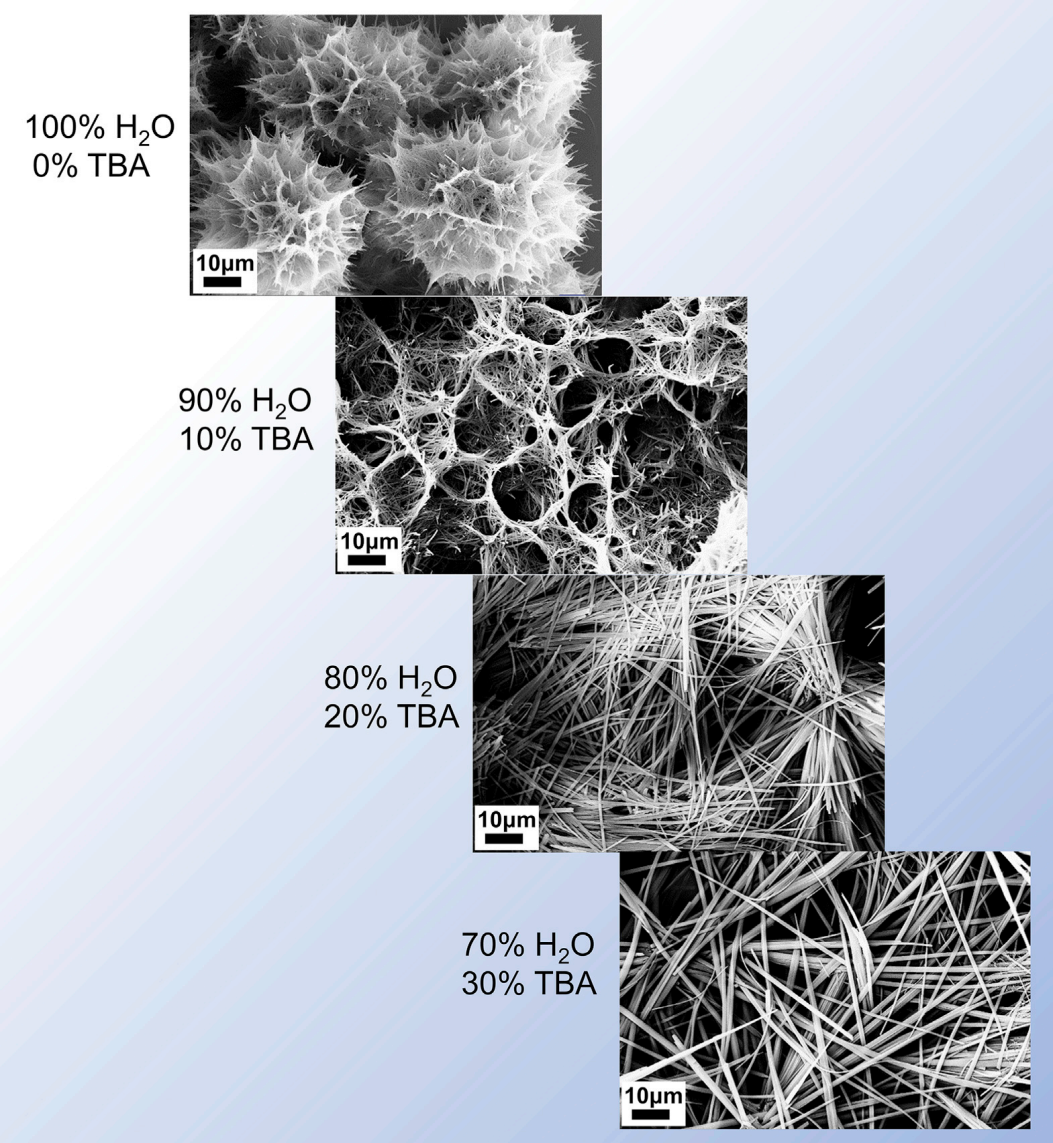
Evolvement of the organization pattern of supramolecular gels with the adjustment of initial solvents

The understanding of the aforementioned phenomena lies in the appreciation of the fundamental principle in supramolecular assembly. Traditional molecular chemistry is mainly concerned with the chemical reaction between different molecules, which involves the break and formation of chemical bonds, whereas supramolecular chemistry basically focuses on non-covalent bonds, i.e., physical interactions between molecules, which allows the assembly of molecules by virtue of the match of physical bonding sites (Lindoy, 2000). Rather than chemical bonds—once formed then hardly changed, the driving forces of the supramolecular assembly are physical interactions—flexible enough to yield varied assembly patterns and easily disturbed to be reversible. As demonstrated before, three raw molecules, M, M∗, and B, are able to assemble together via hydrogen bonding, which is among supramolecular interactions. Conceivably, intervention in the interactions between molecules has influence on the assembly pattern and then the micromorphology of supramolecular gel—this chain can spread to the inner organization of BN aerogel and the attendant properties. The adjustment of solvent here functions as an interference in assembly process. To trace how it works, a series of observations via scanning electronic microscopy (SEM) were conducted to gain more details (Figure 2). Evidently, as the proportion of TBA increased, the micromorphology of supramolecular gel assembled from M, M∗, and B could evolve gradually from a flower-like hierarchy to a flatten pattern, implying that such solvent-based strategy is exquisite enough for microstructure regulation, the wisdom behind which may also inspire other attempts to fabricate engineerable materials.

**Figure 3:**
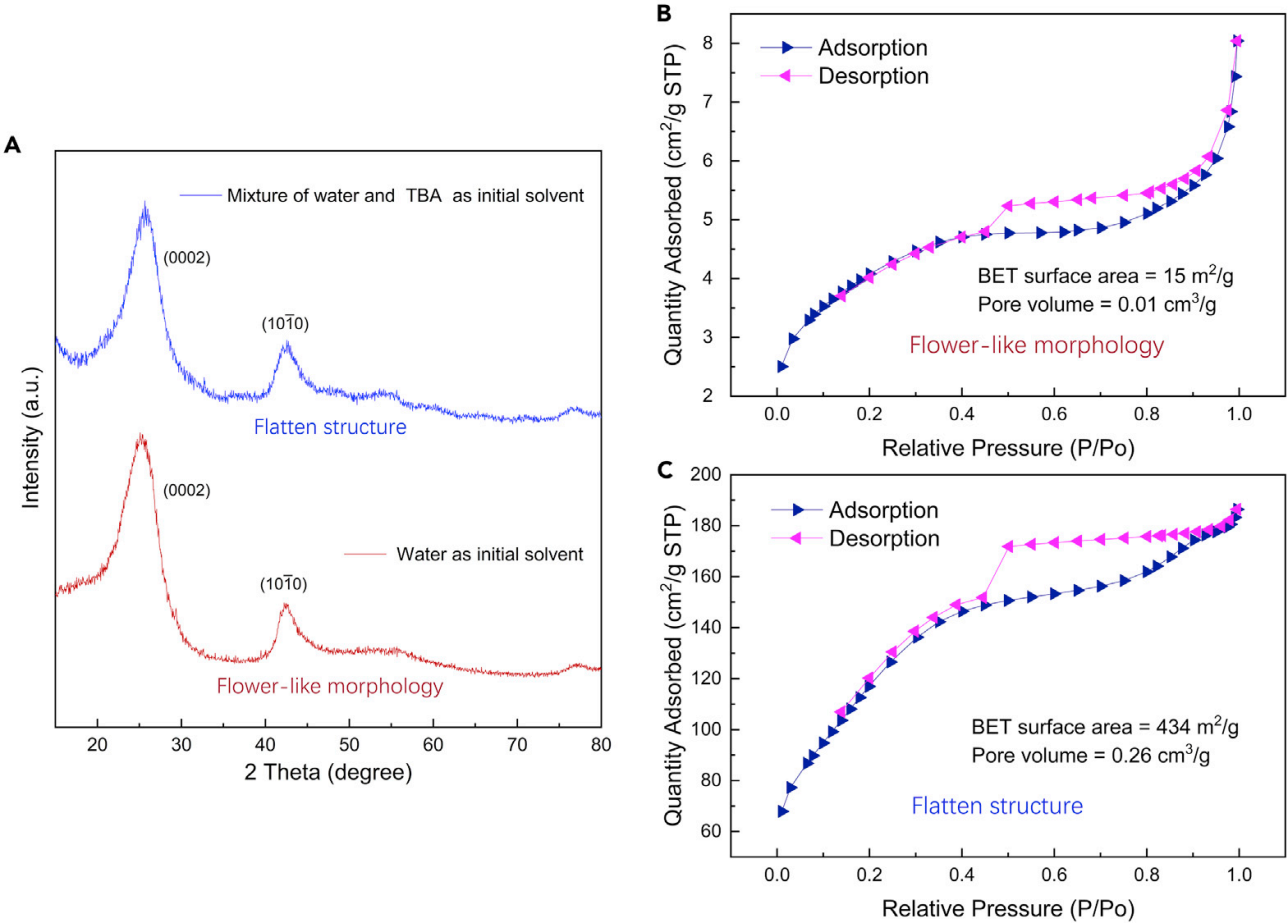
XRD characterization and N_2_ adsorption tests on BN aerogels (A) XRD patterns of the resultant BN aerogels obtained in different cases. (B and C) N_2_ adsorption results of the as-prepared BN aerogels.

After a conversion process under high temperature, the final BN aerogels were obtained. BN aerogel inheriting a flower-like morphology has a density of 16.86 mg/cm^3^ (porosity: 99.25%); the other sample with a flatten structure has a density of 16.75 mg/cm^3^ (porosity: 99.26%), and the summary of these details can be found in supplemental information (Table S1). To confirm the phase composition, X-ray diffraction (XRD) characterization was employed and the characteristic peaks arising from (0002) and (10$\bar{1}$0) planes of hexagonal BN (h-BN) are clearly seen (Figure 3A), and the broad peaks indicate the presence of turbostratic BN (t-BN), which is less crystalline and has a larger interlayer distance than h-BN (3.34 Å). On the whole, the XRD patterns of two samples are quite similar. Transmission electron microscopy (TEM) observation further reveals the detailed microstructure. Compared with that of the other sample derived from supramolecular precursor gel formed in the mixture of water and TBA, the lattice fringes of the BN aerogel with water as initial solvent are a bit less ordered and have a slightly larger spacing (Figure S2). Despite the little difference in TEM results, the adjustment of starting solvents overall has insignificant effect on phase composition of the resultant aerogels. Furthermore, nitrogen (N_2_) adsorption tests were also conducted, and the test results are displayed in Figures 3B and 3C. We can see that the as-prepared BN aerogels in different cases show different N_2_ adsorption patterns. Also, the calculated surface areas based on Brunauer–Emmett–Teller (BET) model are quite different, which reveals the contrast in micro- and mesopore range.

### BN aerogels with different structural hierarchy display similar thermal insulation

Traditionally, aerogel is perceived as a superlight material being of extremely low thermal conductivity, which has overwhelming advantages in fields requiring super thermal insulation (Hu et al., 2019; Illera et al., 2018; Tychanicz-Kwiecień et al., 2019). Contrary to the general aerogel, BN per se is well known for its brilliant thermal conductivity (Sharma et al., 2020). When the two extremes are combined, which side would the integrated BN aerogel stand? And to what extent would the thermal conductivity of such a unique aerogel get influenced by its inner organizations? Although recent works offer some information about the former question (Lin et al., 2017; Xu et al., 2019), few attempts have been made regarding the latter one.

To explore this, we performed thermal conductivity tests. The results show that BN aerogel with complex hierarchy exhibits a thermal conductivity (κ) of 0.0278 W/(m·K) and BN aerogel with a flatten microstructure has a κ of 0.0263 W/(m·K). The thermal conductivity test provides two pieces of information: that BN aerogel has a considerably low thermal conductivity as general aerogels, such as oxide aerogels (Wei et al., 2007; Zu et al., 2011) and polymer-based aerogels (Fischer et al., 2006; Wang et al., 2019) (Table 1), and that BN aerogels with noticeable difference in their inner organization are similarly thermal insulative—κ of samples count same value, albeit slightly different.

**Table 1:** Comparison of thermal conductivity between different aerogels

Aerogel type	Thermal conductivity (Room temperature, atmospheric pressure)	Reference
SiO_2_ aerogel	0.036 W/(m·K)	(Wei et al., 2007)
Al_2_O_3_ aerogel	∼0.028 W/(m·K)	(Zu et al., 2011)
Polyvinylpolymethylsiloxane aerogel	∼0.022 W/(m·K)	(Wang et al., 2019)
Cellulose-based aerogel	0.029 W/(m·K)	(Fischer et al., 2006)
BN aerogels (with complex hierarchy)	0.0278 W/(m·K)	This work
BN aerogels (with flatten structure)	0.0263 W/(m·K)	This work
SiC aerogel	0.026 W/(m·K)	(Su et al., 2018)
Graphene aerogel	∼0.1 W/(m·K)	(Fan et al., 2014)
Carbon aerogel	∼0.026 W/(m·K)	(Feng et al., 2011)

Evidently, when existing in the form of aerogel, BN becomes extrinsically thermal insulative despite its intrinsically high thermal conductivity, implying that the highly porous feature of aerogel counts much more than the intrinsic characteristic of the skeleton on determining the thermal conductivity of an aerogel. The same holds true for other aerogels whose skeletons consist of highly thermal conductive constituents (Table 1), such as silicon carbide (SiC) aerogel (κ = 0.026 W/(m·K)) (Su et al., 2018), graphene aerogel (κ∼0.10 W/(m·K)) (Fan et al., 2014), and polymer-derived carbon aerogel (κ∼0.026 W/(m·K)) (Feng et al., 2011). The values of κ in this work are also approximate to those of other BN architectures reported recently, such as BN foam derived from precursors (∼0.035 W/(m·K)) (Lin et al., 2017) and BN aerogel fabricated via replicating graphene aerogel template (∼0.020 W/(m·K)) (Xu et al., 2019). Furthermore, the fact that hierarchy reduction of BN aerogel has little influence on its apparent thermal conductivity implies that the effect of merely tailoring microstructure of the skeleton may be insignificant when one seeks to substantially tune the thermal conductivity of an aerogel at a certain porosity. On the other level, it may also benefit researchers devoted to thermal management (Liu et al., 2020).

### Reduction of structural hierarchy shifts the wettability from hydrophobicity to hydrophilicity

Contrary to the circumstance of thermal conductivity, the affinity of BN aerogel for water is considerably sensitive to the change in structural hierarchy. When inheriting a flower-like morphology, BN aerogel shows hydrophobicity—the contact angle for water is 143° (Figure S3), and this value is near that of BN nanotubular architectures synthesized by template-assisted chemical vapor deposition (CVD) (Xue et al., 2017); by contrast, with a flatten microstructure, BN aerogel displays hydrophilicity—the contact angle for water is 0° (Figure S3), and this value is similar to that of hydroxyl-functionalized BN nanostructures (Pakdel et al., 2014).

It is well-documented that materials' wetting behavior is basically determined by two factors—chemical composition and surface geometry (Feng and Jiang, 2006). The modulation of wettability relies on changing either or both of them. For example, Pakdel et al. have made a series of attempts to tailor the wettability of BN nanostructures by applying chemical functionalization through plasma treatment (Pakdel et al., 2014) or by changing their morphology via adjusting CVD parameters (Pakdel et al., 2013).

In this work, BN aerogels with discrepant inner organizations originated from same raw molecules at fixed molar ratio. Compared with the contribution of surface morphology to wetting behavior, the effect of chemical composition pales into insignificance.

X-ray photoelectron spectroscopy (XPS) analyses further confirm that both BN aerogels show similar surface chemical states and their surfaces are rich in hydrophilic species. XPS full scan spectra (Figure S4) identify four elements—B, N, C, and O, the 1s peaks of which are observed at around 190.3, 398.0, 284.6, and 531.8 eV, respectively. For comparison, commercial BN powder is used as a reference. B and N are the main elements in individual samples, whereas C and O may come from the adsorbed carbon dioxide or other species in the air. To gain more details, the narrow scan spectra of B 1s and N 1s are also investigated.

**Figure 4:**
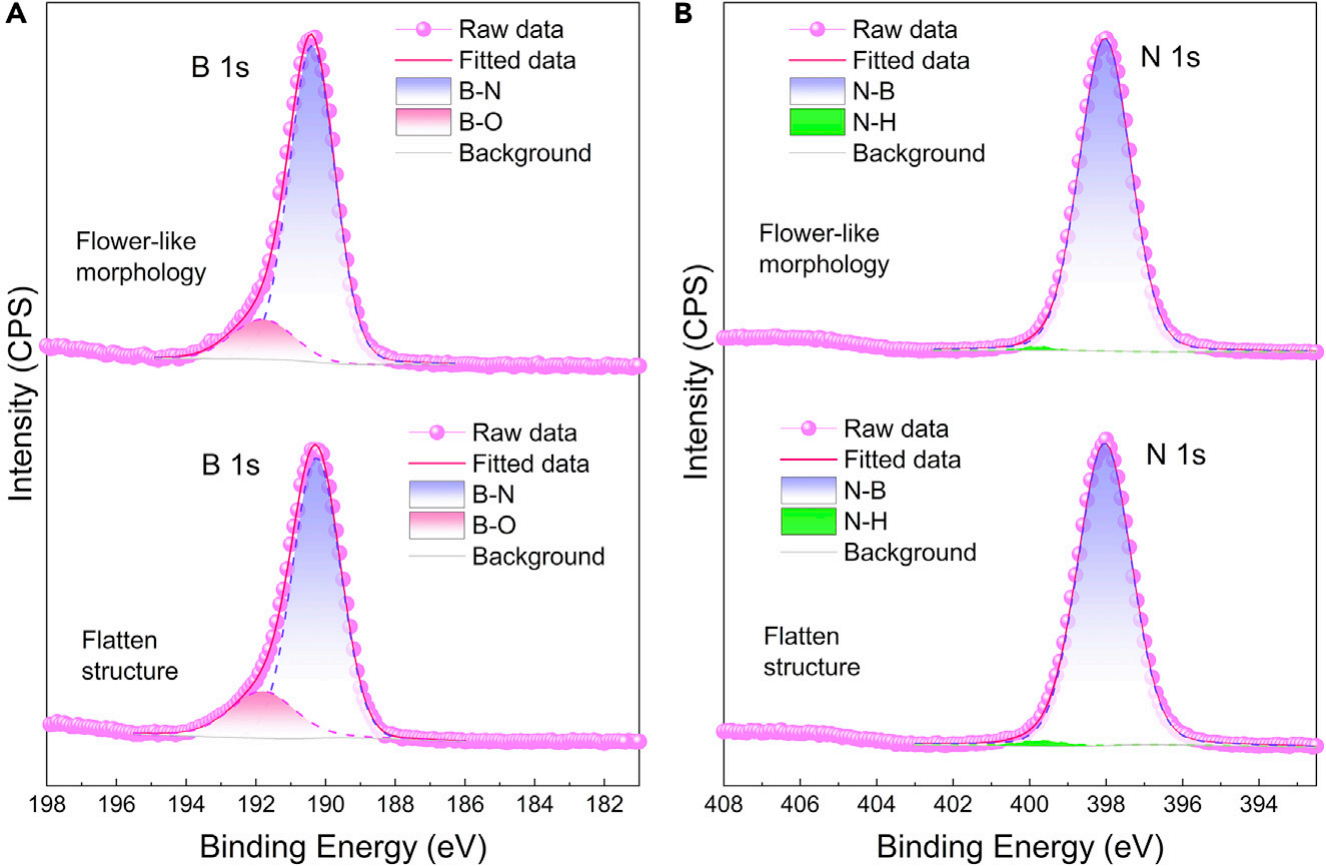
XPS spectra of the as-prepared BN aerogels with different inner organizations (A) B 1s narrow scan spectra; (B) N 1s narrow scan spectra.

As shown in Figure 4, the main peaks corresponding to B-N bonds are located at 190.3 eV in B 1s and 398.0 eV in N 1s. Both B 1s spectra have a shoulder peak fit at 191.8 eV arising from B-O species; likewise, both N 1s spectra have a tiny peak fit at 399.6 eV, which can be assigned to N-H. Overall, two BN aerogels with disparate inner organizations display almost same surface chemistry, which lends weight to the conclusion that the difference in structural hierarchy is the very factor that contributes to the contrast in wettability.

**Figure 5:**
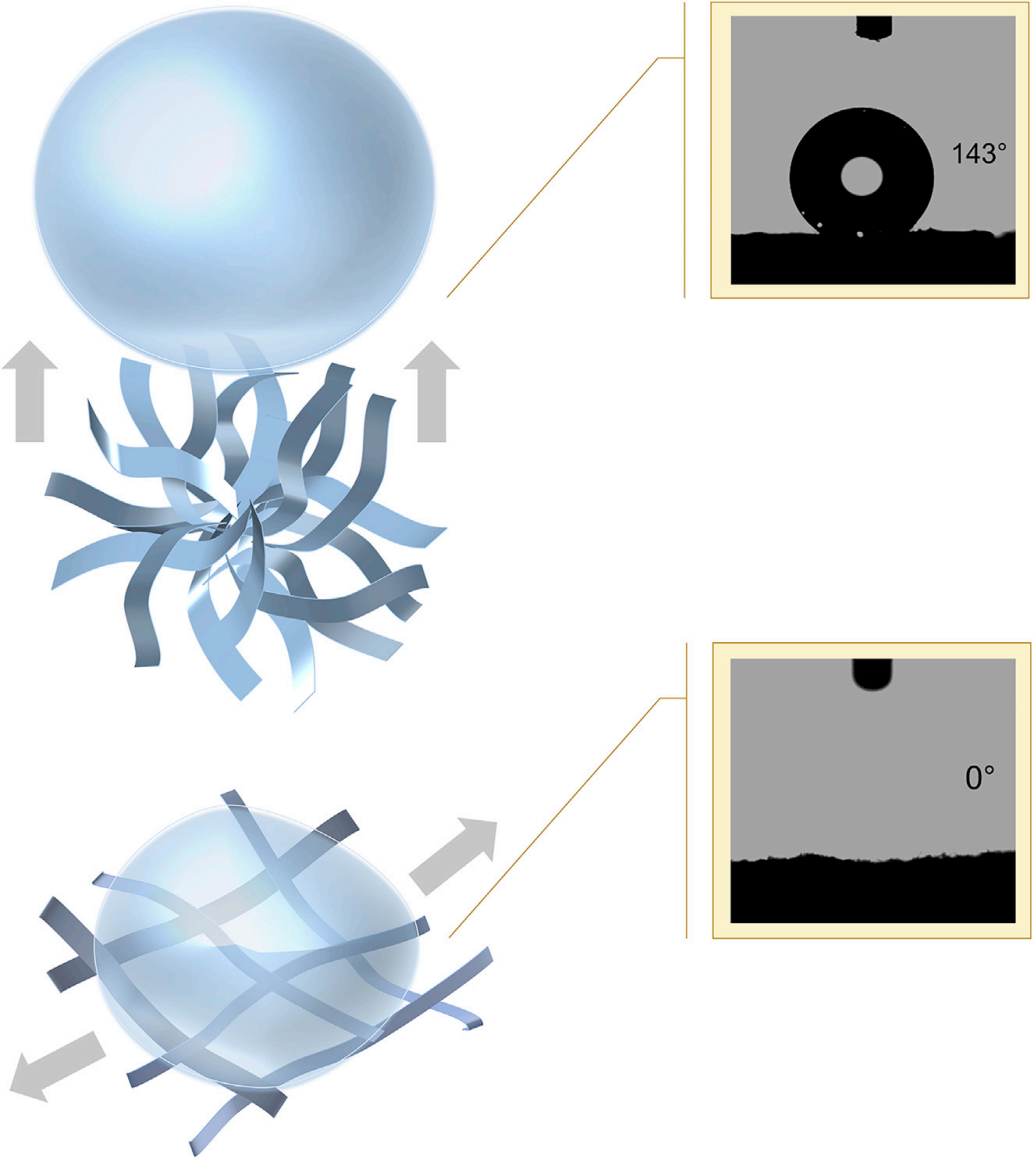
Illustration of wettability contrast led by the difference in structural features

Figure 5 illustrates how surface geometry affects the water affinity of BN aerogels in this work.

When a water droplet gets close to BN aerogel with flower-like hierarchy, the unique structure provides scarce contact points and vapor pockets are trapped between the liquid and solid. Thus, the droplet could easily suspend on the aerogel. Although there lies some hydrophilic species on the surface, the contact area is too small to impact the hydrophobicity.

In terms of the other aerogel, the flatten structure facilitates its contact with the water droplet, which could easily fill the gap between adjacent ribbons. What is more, the hydrophilic surface groups further contribute to the spreading of water. In addition, the three-dimensional porous network enables a 3D capillary effect that is favorable for liquids to intrude into the textured materials spontaneously (Drelich et al., 2011; Tian et al., 2014).

### Change in structural hierarchy leads to great contrast in ion adsorption

Besides the thermal conductivity and wettability, we also sought to investigate the possible effect that reduction in structural hierarchy may have on the adsorption performance for heavy metal ions. Herein, lead ions (Pb^2+^), one of typical metal pollutants in water (Channegowda, 2020), were chosen as a model.

**Figure 6:**
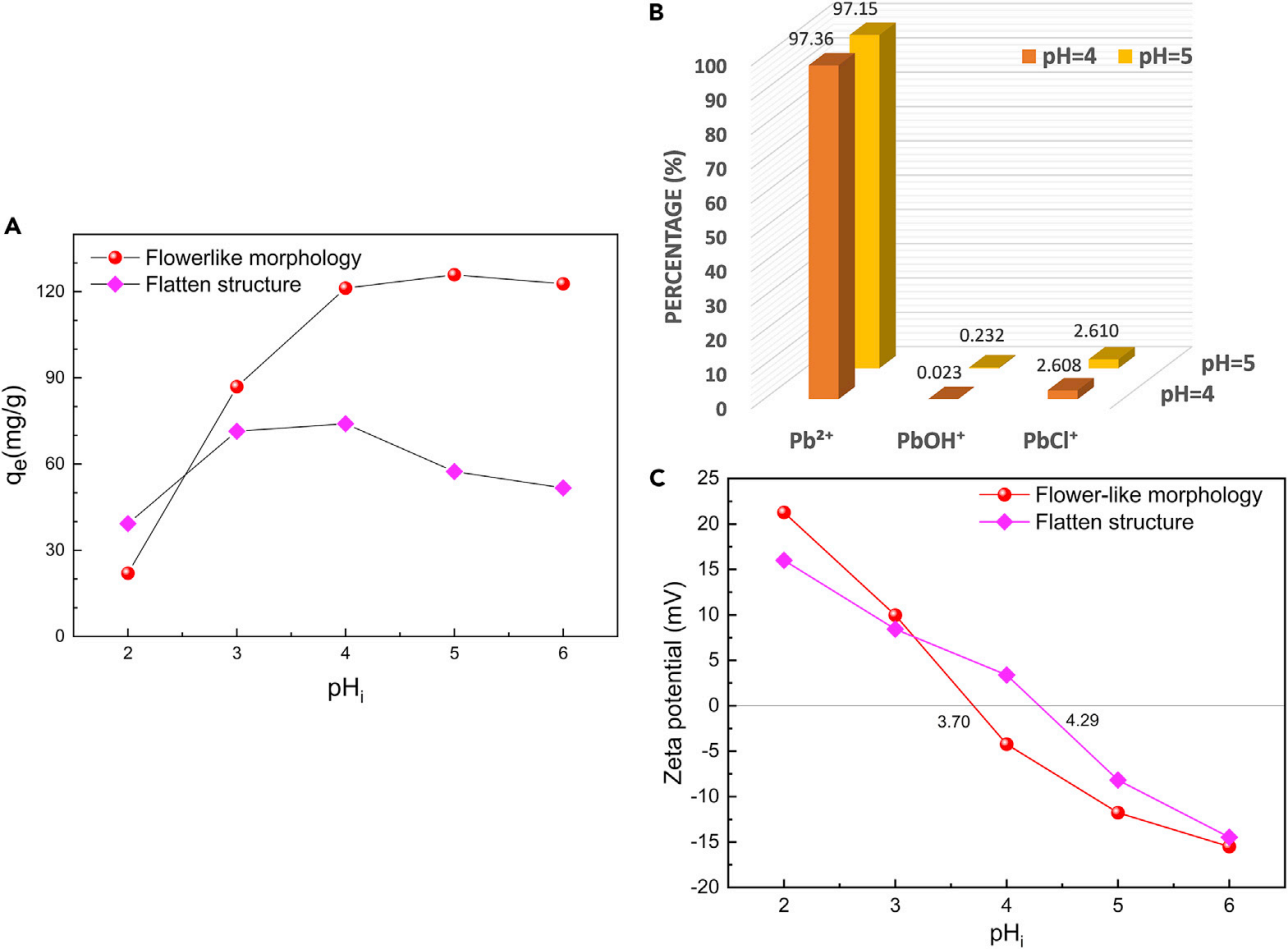
Primary explorations on the adsorption performance of BN aerogels for lead species. (A) Influence of initial pH (pH_i_) on the adsorption capacity of BN aerogels with different inner organizations. (Initial concentration of Pb^2+^: 90 mg/L; volume of PbCl_2_ solution: 30 mL; mass of adsorbent: 0.02 g.) (B) Simulated lead species distribution in PbCl_2_ solution under optimal pH conditions for adsorption in two cases. (C) Zeta potential results of two BN aerogels at different pH conditions.

In the first step, a series of attempts were made to determine the optimal pH condition, given that pH generally has an appreciable effect on the adsorption of heavy metal ions (). As exhibited in Figure 6A, the adsorption performance of two aerogel adsorbents with different inner organizations changes as the initial pH (pH_i_) varies. For BN aerogel inheriting a flower-like morphology, the optimal pH for adsorption is determined to be 5, whereas the optimal pH is 4 for the other aerogel with flatten microstructure.

The aforementioned results primarily show that the change in structural hierarchy greatly impacts the ion adsorption performance of BN aerogels. We conducted further studies to gain more knowledge about this phenomenon.

Commonly, the difference in adsorption results can hardly decouple with two sides, ions and adsorbents. To achieve a good understanding, it is essential to figure out the states of both sides.

To know the states of Pb under optimal pH conditions, we simulated the distribution of lead species in all pH range (Figure S5) and summarized the species distribution under two optimal pH, i.e., 4 and 5, in Figure 6B. As can be seen, there are three types of Pb(Ⅱ) species existing in the above-mentioned solutions for adsorption. Their proportion follows the order Pb^2+^ > PbCl^+^ > PbOH^+^, among which Pb^2+^ ions are the dominant species.

Furthermore, we conducted the zeta potential tests to examine the surface charge of adsorbents. As marked in Figure 6C, the isoelectric points (pI) of two BN aerogels are different. BN aerogel with flower-like hierarchy has a pI of 3.70, which is lower than the figure for the other aerogel with flatten structure (4.29). At optimal pH conditions for adsorption, the former adsorbent is negatively charged, facilitating the electrostatic attraction of lead species, whereas the latter is electrically positive on its surface, and the electrical interaction in this case contributes little to the adsorption process and even has counter-effect.

**Figure 7:**
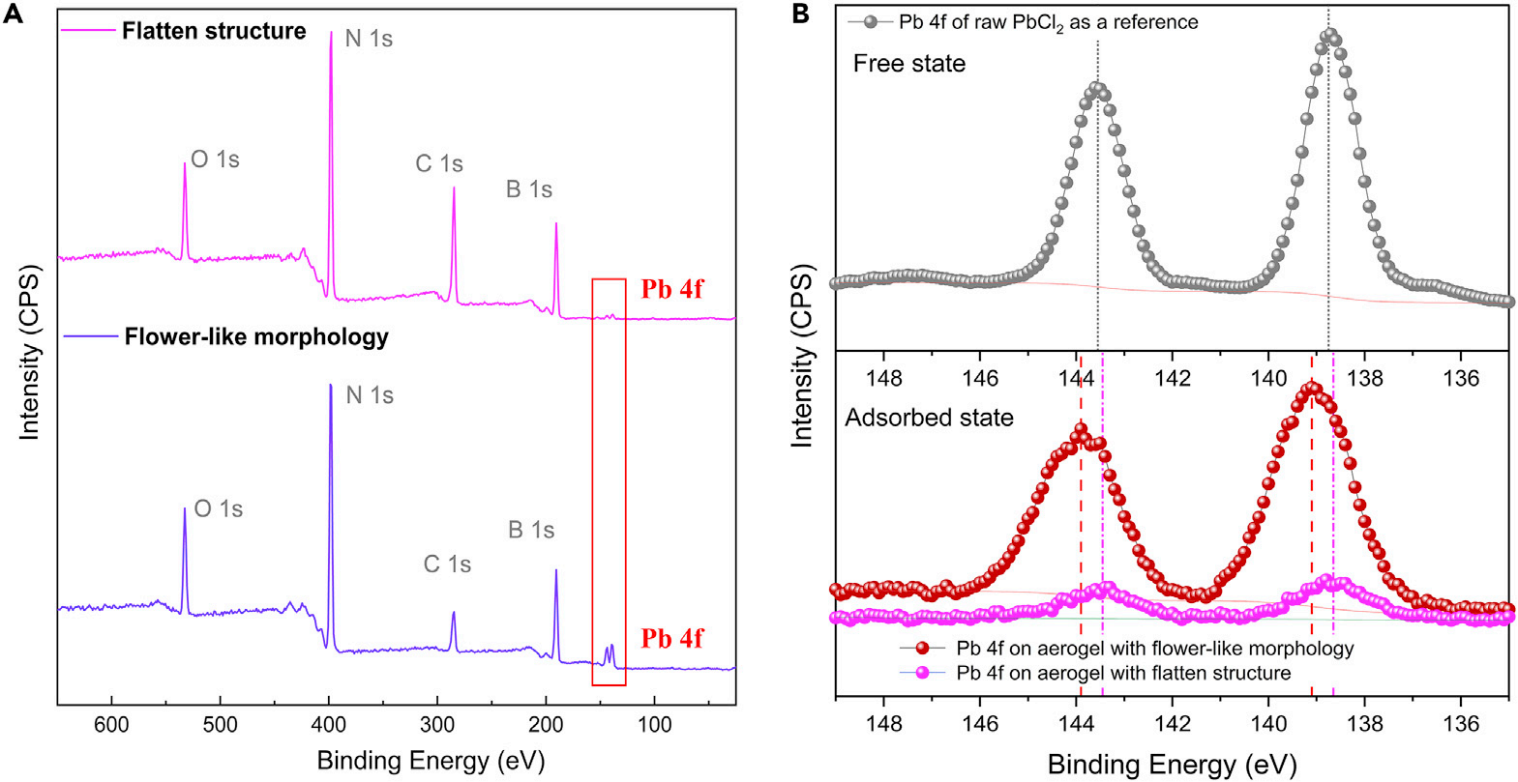
Surface chemical states of BN aerogels after the adsorption of lead species. (A) XPS full scan spectra of two BN aerogel adsorbents after the adsorption of lead species; (B) XPS narrow scan spectra of Pb 4f in different cases.

In addition, XPS analyses were performed to investigate the surface states of BN aerogels after they absorbed lead species from PbCl_2_ solution under optimal conditions. When compared with the original XPS survey spectra shown in Figure S4, two new peaks corresponding to Pb 4f are clearly to be seen in Figure 7A, indicating the successful adsorption of lead species on the surface of two aerogels. The disparity in the intensity of Pb 4f peaks can be attributed to the difference in adsorption amount.

We further studied the XPS narrow scan spectra to gain more insight into the adsorption mechanism. The upper part of Figure 7B shows Pb 4f peaks for the free state of lead ions, whereas the lower part displays Pb 4f signals detected on the surface of two aerogel adsorbents after the adsorption of lead species.

In terms of BN aerogel derived from flower-like morphology, the detected Pb 4f signal shows a noticeable shift (+0.4 eV) to higher binding energy; accordingly, a negative shift accompanied by an increase in intensity can be observed in signals of B-O and N-H species (Figure S6), implying a strong chemical interaction between the adsorbent and lead species.

When it comes to BN aerogel with flatten structure, the detected Pb 4f signal shifts inappreciably to lower energy (−0.05 eV), which pales into insignificance when set against that in the other circumstance. In the narrow scan spectra of B 1s (Figure S7), we can see that the shoulder peak corresponding to B-O species shifts slightly to lower binding energy and its intensity decreases after the adsorption of lead ions. A reasonable explanation is that at low pH, B-O species tend to interact with H^+^ and form B-OH at first; then the B-OH species connect with lead ions via surface complexion, which leads to a decrease in the peak intensity of B-O species. In N 1s narrow scan spectra (Figure S7), it can be seen that N-H signal shows a slight upward shift compared with the pristine state, implying that N-H may interact with lead species via surface complexion, which results in reduced electron density in N atoms after the adsorption process.

From the XPS results, it is known that there lies appreciable chemical interaction between lead species and BN aerogel of complex hierarchy; whereas for the other aerogel of flatten structure, such chemical interaction is far less significant than that in the former case.

**Figure 8:**
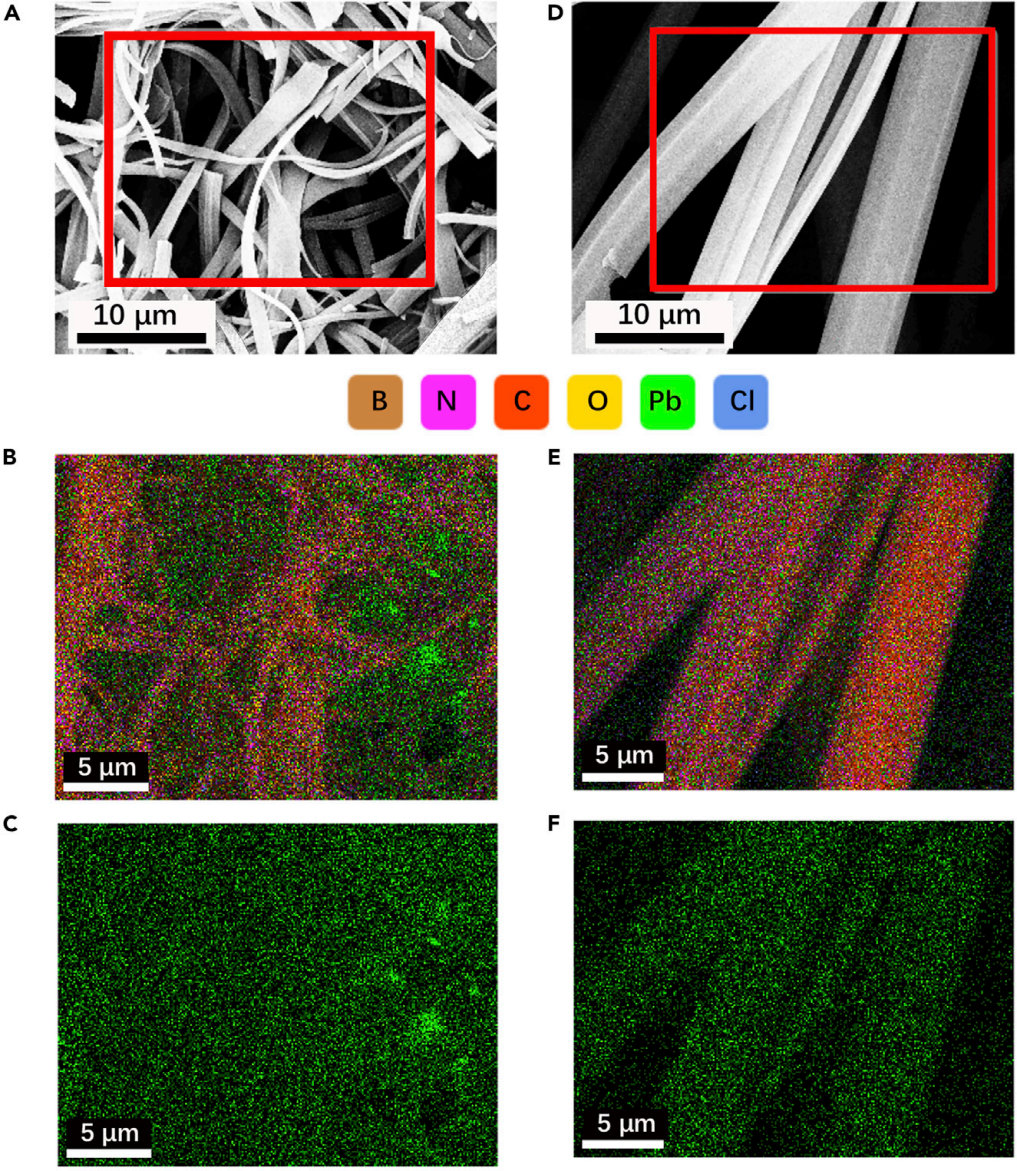
EDS analyses for BN aerogels after the adsorption of lead ions (A and D) SEM images of two BN aerogels after adsorption experiments. Red frames mark the selected regions for EDS mapping. (B and E) Overall mapping overlaid by different elements, corresponding to (A) and (D). (C and F) Individual mappings of lead element.

To get a clear picture of the distribution of the adsorbed lead ions within different structures, we further conducted EDS analyses and compared the results in Figure 8.

It is evident that the heavy metal element, lead (Pb), which is marked by green, is much more conspicuous within BN aerogel of complex hierarchy (Figure 8B), whereas it is unobvious within BN aerogel inheriting a flatten pattern (Figure 8E).

Based on the aforementioned results, we present here a reasonable explanation about the cause for the distinct difference in adsorption results.

**Figure 9:**
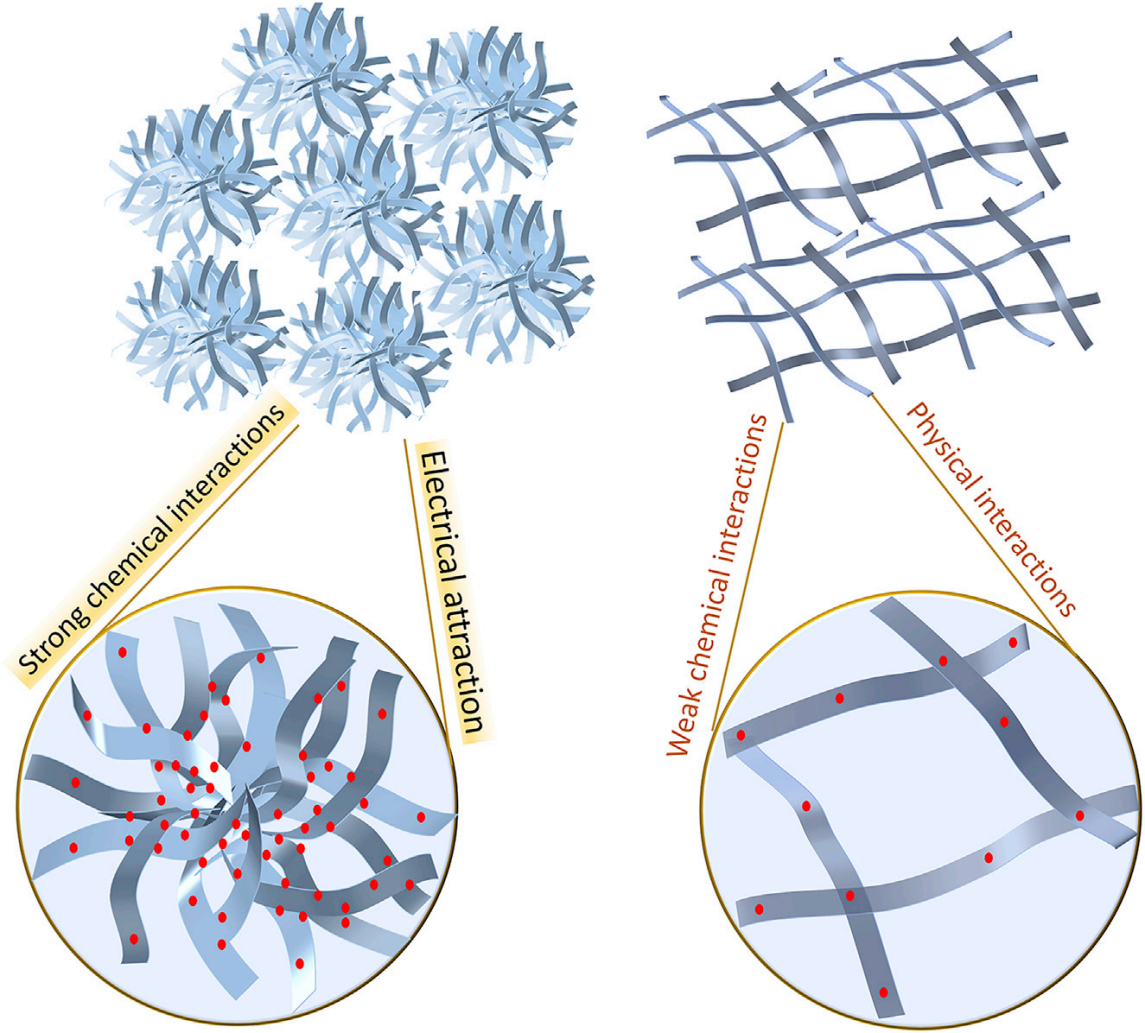
Illustration of the adsorption of lead species within a complex hierarchy and a flatten structure

As illustrated in Figure 9, within the flower-like hierarchy, there lie various tiny cells, providing localized environment for heavy metal ions to reside and interact sufficiently with the adsorbent; therefore, the lead ions are able to anchor substantially in such areas and form strong chemical interactions with the aerogel skeletons. In addition, electronic attraction also facilitates the adsorption process. By contrast, the flatten pattern is not favorable for retaining metal species, and the chemical interaction between aerogel adsorbent and lead ions is inappreciable. At the optimal condition, the electronic interaction is repulsive in this circumstance and unfavorable for the adsorption process. And it can be speculated that physical interactions such as van der Waals force also play a part.

Subsequently, the correlation between time and removal rate as well as ratio of residual concentration of Pb^2+^ were also studied.

**Figure 10:**
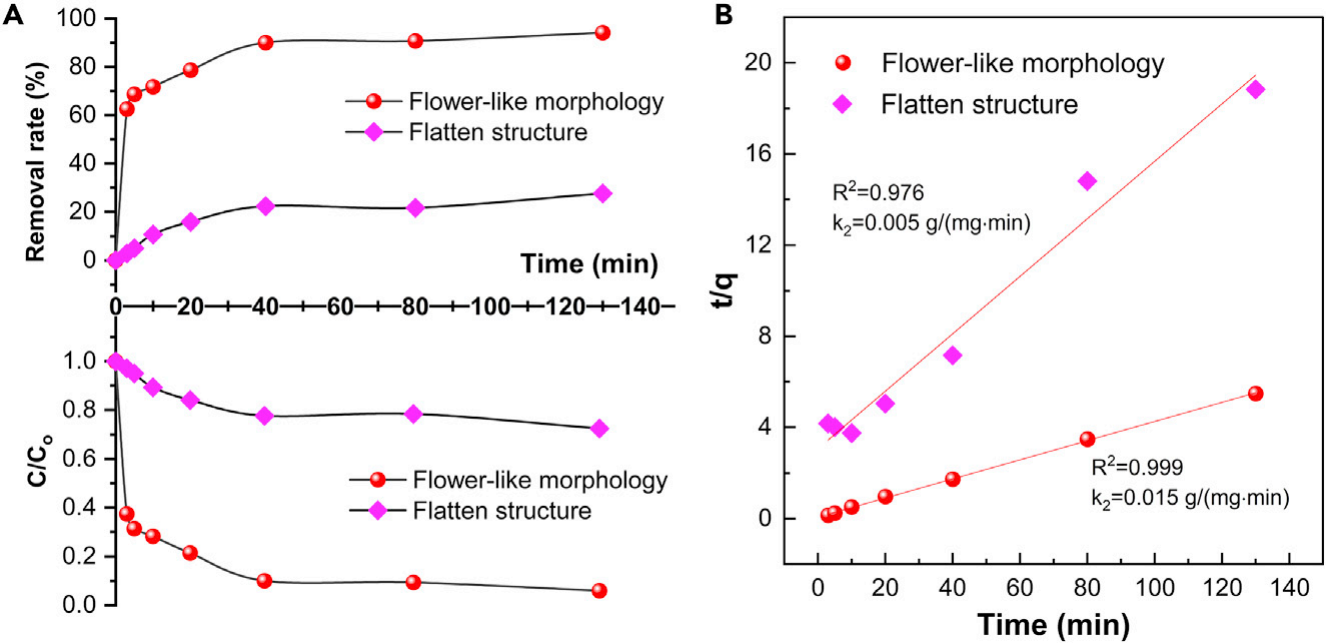
Kinetic studies on two adsorption processes. (A) Correlation between time and removal rate as well as ratio of residual concentration of lead ions; (B) Pseudo-second-order fitting of the adsorption processes in two cases.

As plotted in Figure 10A, the residual Pb^2+^ ratios in both cases show a downward tendency and gradually get to a stable level with the increase of contact time. Noticeably, the ratio of C/C_o_ in terms of BN aerogel derived from flower-like morphology reaches nearly 0, whereas that for the other aerogel with flatten microstructure is no less than 0.7, indicating a great contrast in adsorption performance. When it comes to removal rate, we can see that the figure for the first aerogel sample outnumbers that for the second one in all time range. The removal rate of aerogel with flower-like organization peaks at over 90%, which is three times more than that of the other aerogel with flatten structure, accounting for no more than 30%.

To further study the adsorption kinetics, two classical models are involved. First, pseudo-first-order model is used for the fitting of the adsorption of leads ions. However, it does not match well with the practical adsorption process in this work, i.e., the coefficient of determination (R^2^) is less than 0.9, as summarized in Figure S8.

When compared with the first fitting, pseudo-second-order model fits satisfactorily with the adsorption processes, indicating the existence of chemisorption in both cases (Figure 10B). Based on the fitting results, the adsorption rate constant k_2_ can be deduced. It can be known that the adsorption processes ran at different rates, as the rate constants have a 3-fold difference in their values.

**Figure 11:**
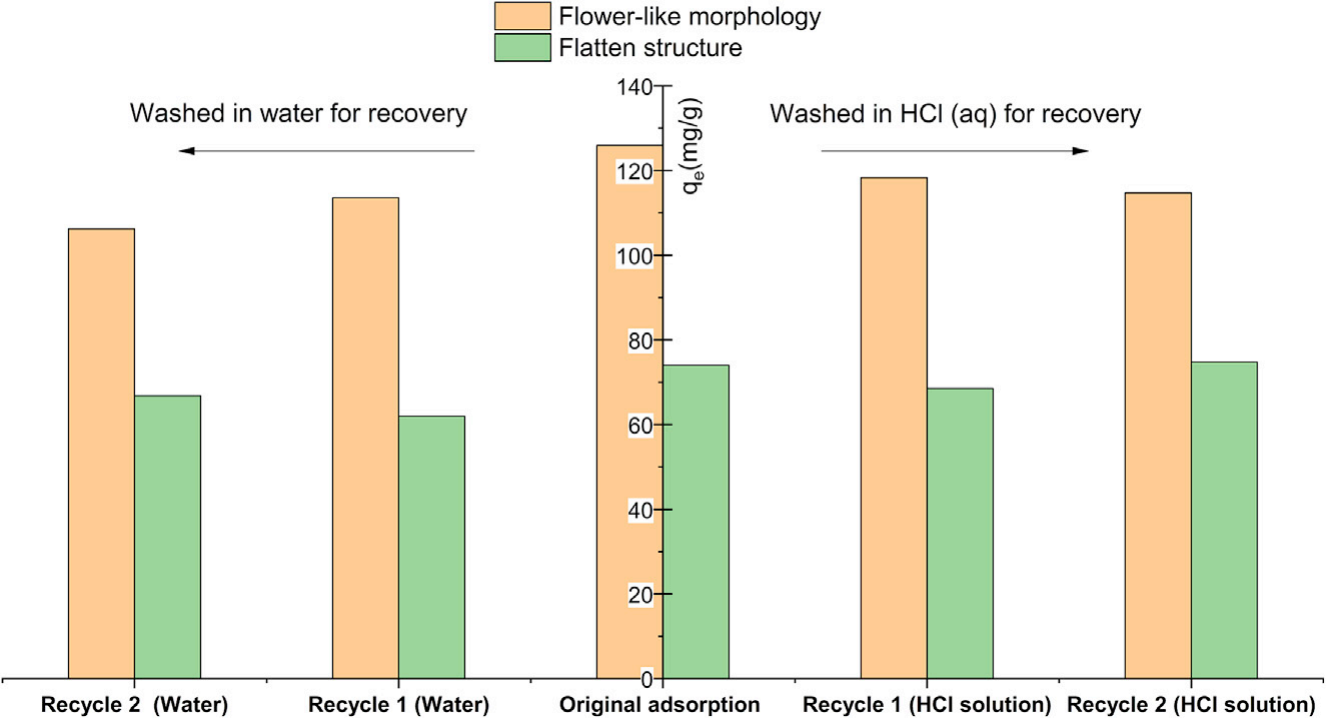
Regeneration of two BN aerogel adsorbents under different recovery conditions Parameters for adsorption experiments: initial concentration of Pb^2+^: 90 mg/L; volume of PbCl_2_ solution: 30 mL; pH: optimal values; mass of adsorbent: 0.02 g.

Furthermore, we conducted the regeneration experiments, during which different post-treatments were applied. In one case, two aerogel adsorbents were washed by distilled water for recovery; in the other case, they were washed in hydrochloric acid (HCl solution). Figure 11 shows a summary of adsorption results when the two BN aerogels were regenerated twice. The original adsorption result is also displayed here for reference.

In the same recycle round, aerogel adsorbents recovered in HCl solution display better adsorption capacity than the same samples recovered in water, indicating that post-washing in acid solution contributes to better recovery efficiency than the same treatment in water.

Another difference lies in the contrast in adsorption capacity tendency. For BN aerogel with flower-like hierarchy, the adsorption capacity tends to go downward in both regeneration conditions. For the other with flatten structure, the adsorption performance could be recovered near to the original state, albeit it drops a little in the initial recycle round.

On the whole, BN aerogel with complex hierarchy outperforms the other of flatten structure in all recycle adsorption tests. The difference in recovery degree could be ascribed to the interaction between adsorbents and ions. As there lies strong chemical interactions between the former aerogel and lead species, the adsorption capacity of adsorbent in this case downgrades after each run. In comparison, the latter aerogel could be better recovered, as the chemical interaction is insignificant and the physical forces between adsorbents and ions could be easily disturbed.

As has been shown, two types of BN aerogels in this work are derived from same raw molecules with a fixed molar ratio, and they exhibit similar surface chemistry in their pristine states, while they display disparate adsorption capability for lead ions. A flatten microstructure contributes to a low efficiency, whereas a complex hierarchy bestows better adsorption performance upon the material with same chemical composition. Based on this finding, we know that the modulation of adsorption properties of BN nanomaterials can be achieved by the construction of special hierarchy. Compared with the common functionality strategy, this work has some advantages for BN materials, which are usually chemically inert and relatively harder to be functionalized when compared with their carbon counterparts (Ahmad et al., 2020; Bassyouni et al., 2020). This study would pave ways for the application of BN aerogel with special hierarchy in water purification (Ihsanullah, 2021) and pollutant removal (Yu et al., 2018).

To verify that the difference in adsorption performance led by hierarchy contrast also applies to other heavy metal ions. Another two ions, Cu^2+^ and Cr^3+^ were additionally used for the adsorption tests. In these cases, the adsorption performance of BN aerogel derived from flower-like structure excels that of the other aerogel with flatten microstructure under optimal pH conditions (Figure S9), confirming that the complex hierarchy is superior to the flatten pattern in many scenarios.

## Discussion

We are inspired by nature's lesson that different levels of organization and complexity could lead to versatile functions and attempt to explore this principle in a ceramic aerogel system. With the wisdom borrowed from supramolecular world, an exquisite approach is displayed herein for the microstructure tailoring of BN aerogel, a typical inorganic aerogel. Solvent is exploited as a tool to intervene in the assembly process of the supramolecular precursor gel—the simple adjustment of initial solvents influences the assembly pattern of supramolecular gel and then the structural hierarchy of final BN aerogel as well as its attendant properties.

It is noticeable that BN could become extrinsically thermal insulative in the form of aerogel despite its intrinsically high thermal conductivity, whereas the reduction in structural hierarchy has little effect on the apparent thermal conductivity of BN aerogel at a certain porosity, which may benefit researchers devoted to thermal management. Different from the circumstance of thermal conductivity, the wetting behavior of the as-obtained aerogel is considerably sensitive to the discrepancy in inner hierarchy. Interestingly, the hierarchy difference also leads to varied performance for the adsorption removal of heavy metal ions. A flatten pattern contributes to a low efficiency alongside a weak interaction between the adsorbates and skeletons, whereas a complex hierarchy bestows better adsorption performance upon the aerogel with same chemical constituent. Although it is impossible to explore all properties correlated with structural hierarchy in a single article, this work provides a window to the primary understanding about the significance of special hierarchy existing in an intricate ceramic aerogel. Meanwhile, it leaves plenty of space worthy of further explorations, for example, unveiling more interesting phenomena attendant with special hierarchy or constructing versatile architectures for various purposes.

Another implication of this work is that the arts of non-covalent interactions, traditionally working in supramolecular chemistry domain, could be extended to other fields that are not limited to mild circumstances. It is logically possible to follow similar principles and flexibly synthesize more inorganic materials with tailorable hierarchy, which endure extreme conditions and display versatile functions. Benefiting a lot from interdisciplinary lessons, this article may also inspire other perspectives for materials design on the back of the intercourses between different subjects.

### Limitations of the study

This work borrowed the wisdom from supramolecular assembly and applied it in the design of BN aerogels, which were converted from supramolecular gels after a high-temperature treatment. BN aerogel here serves as an example. The extending of the design principle to other materials systems remains to be unexplored.

For the adsorption experiments, this study did not straddle all influencing parameters. The effects of dosage of absorbents, temperature, ion strength, and other related factors are not included. In addition, this work selected only several types of ions for adsorption tests, and thus the utmost capacity of the adsorbents has not been revealed.

### Resource availability

#### Lead contact

Further requests for resources regarding this study will be fulfilled by the corresponding author, Jingyang Wang (jywang@imr.ac.cn).

#### Materials availability

This work did not produce any new unique reagents.

#### Data and code availability

This work did not generate datasets/code.

## Methods

All methods can be found in the accompanying transparent methods supplemental file.

## Supplemental information

Document S1. Transparent methods, Figures S1–S9, and Table S1

## Footnotes

Supplemental information can be found online at https://doi.org/10.1016/j.isci.2021.102251.
