# Supplementary material for: Reduction of structural hierarchy translates into variable influence on the performance of boron nitride aerogel
Source: iScience. 2021 Mar 1;24(3):102251. doi: 10.1016/j.isci.2021.102251 (PMC7973872; doi:10.1016/j.isci.2021.102251)
Supplement: Document S1. Transparent methods, Figures S1–S9, and Table S1 [file mmc1.pdf]

iScience, Volume *24*

Supplemental information

Reduction of structural hierarchy translates into variable influence on the performance of boron nitride aerogel

Jingjing Pan and Jingyang Wang

Supplemental Data

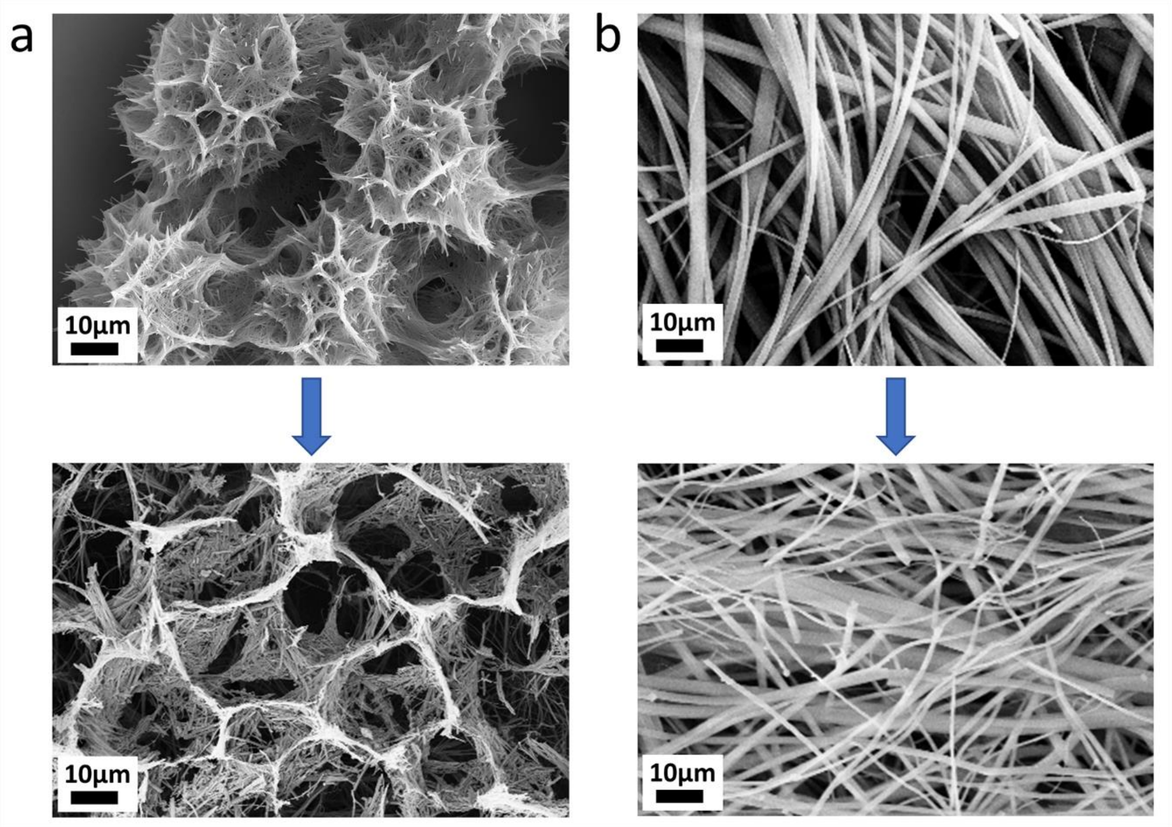

Figure S1 SEM images of supramolecular gels and the corresponding BN aerogels. For (a), the solvent for the formation of supramolecular gel was pure water; for (b), the initial solvent was the mixture of water and TBA (TBA took up 30%), *Related to Figure 1.*

Table S1 Density and porosity of BN aerogel in different cases, *Related to Table 1.*

Initial solvent	Micromorphology of BN aerogel	Density (average value)	Porosity (average value)
Water	Flower-like structure	16.86 mg/cm^3	99.25%
The mixture of water and TBA (TBA took up 30%)	Flatten structure	16.75 mg/cm^3	99.26%

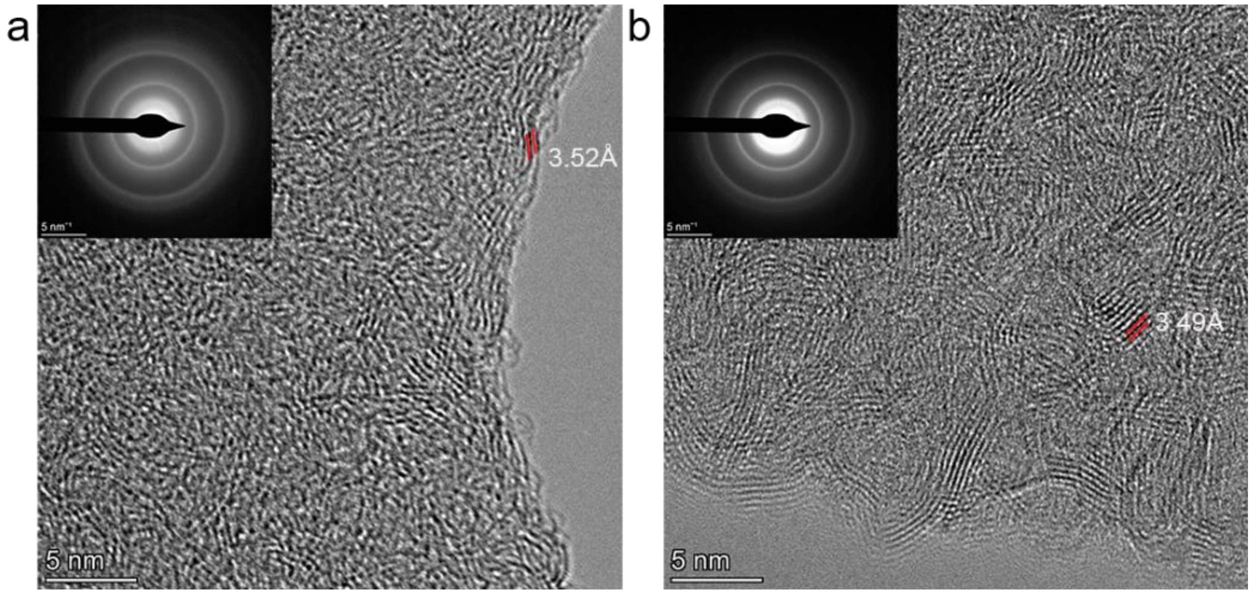

Figure S2 TEM characterization of BN aerogels obtained in different cases. For sample (a), the initial solvent for the formation of its supramolecular precursor gel is water; for (b), the initial solvent is the mixture of water and TBA (TBA took up 30%), *Related to Figure 3.*

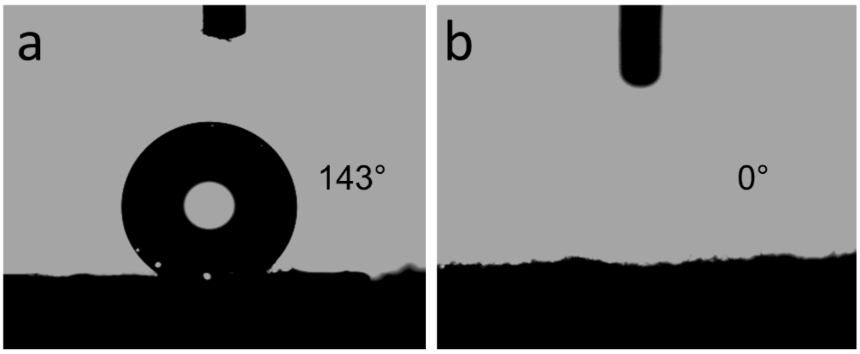

Figure S3 Water contact angle of BN aerogel with flower-like morphology (a) and flatten microstructure (b), *Related to Figure 4 and 5.*

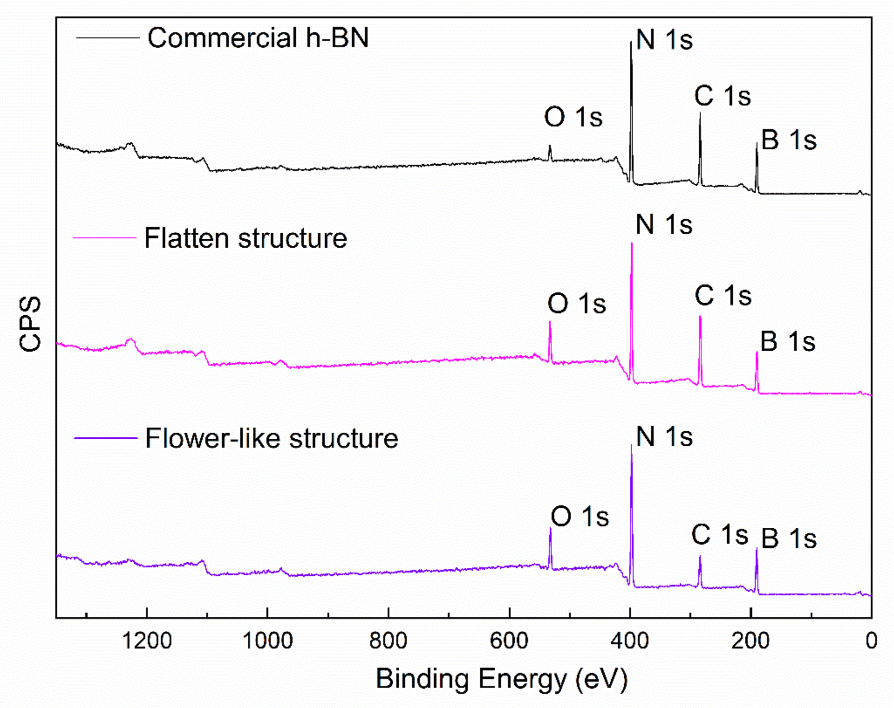

Figure S4 XPS full scan of BN aerogels with different inner organizations. Commercial h-BN is also presented for reference, *Related to Figure 4.*

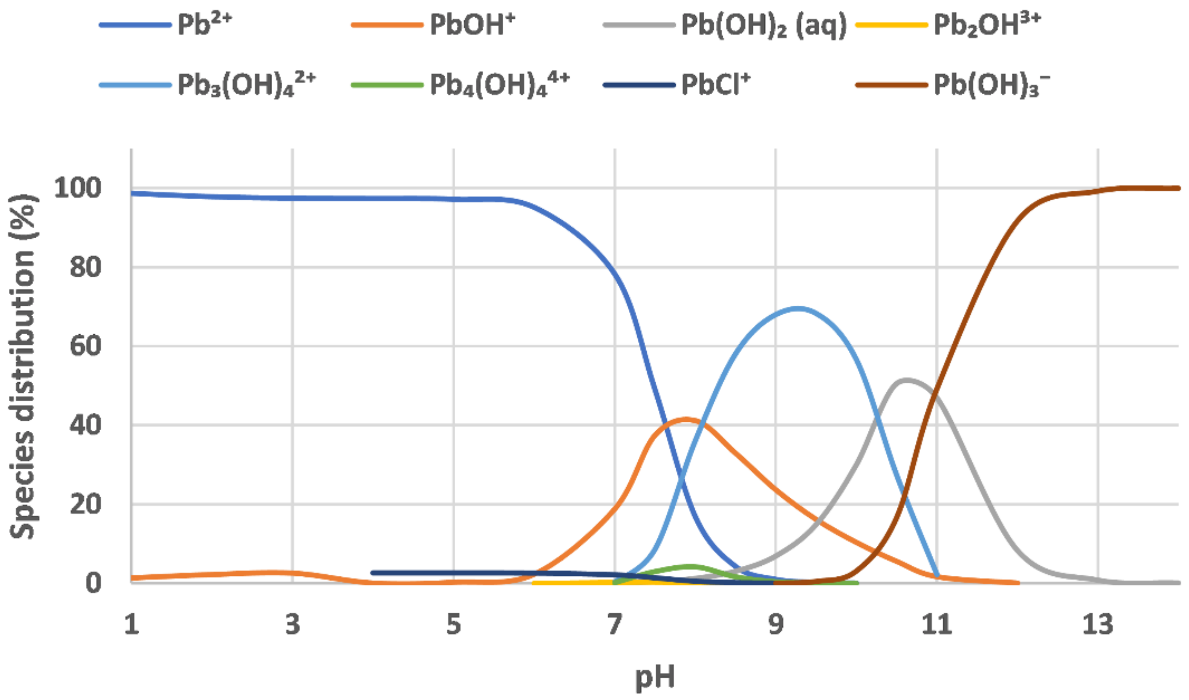

Figure S5 Simulated lead species distribution in $PbCl_2$ solution in all pH range, *Related to Figure 6.*

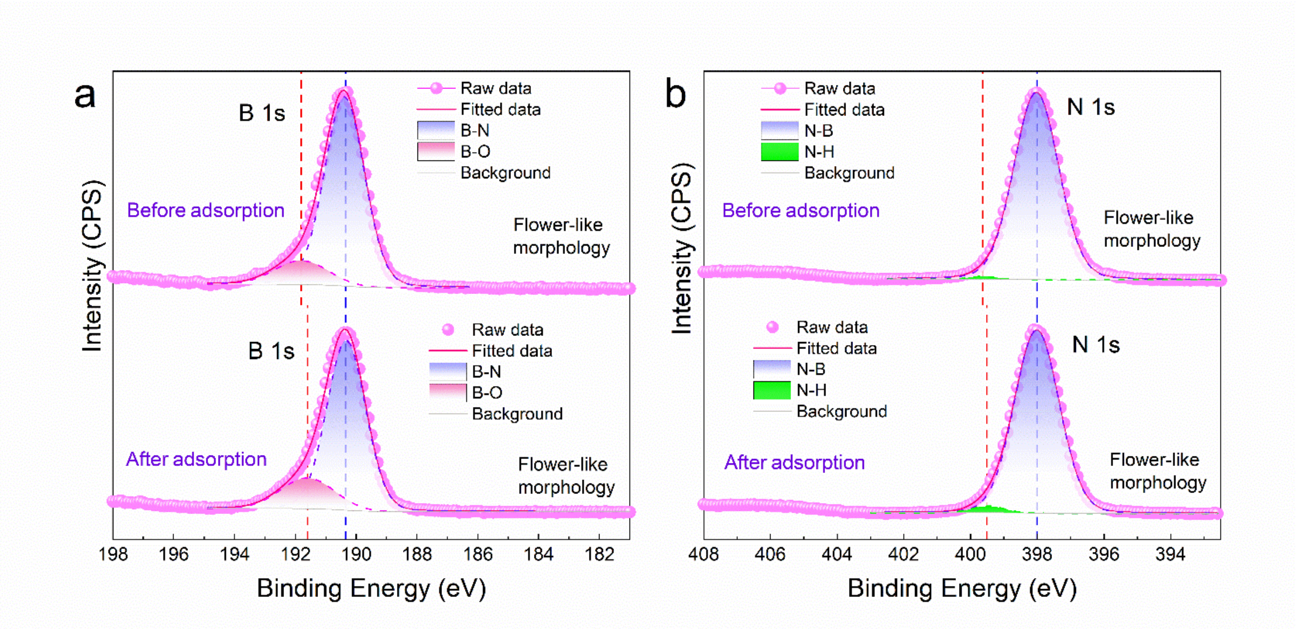

Figure S6 Comparison of XPS narrow scan spectra of BN aerogel inheriting a flower-like organization before and after the adsorption of lead species, *Related to Figure 7.*

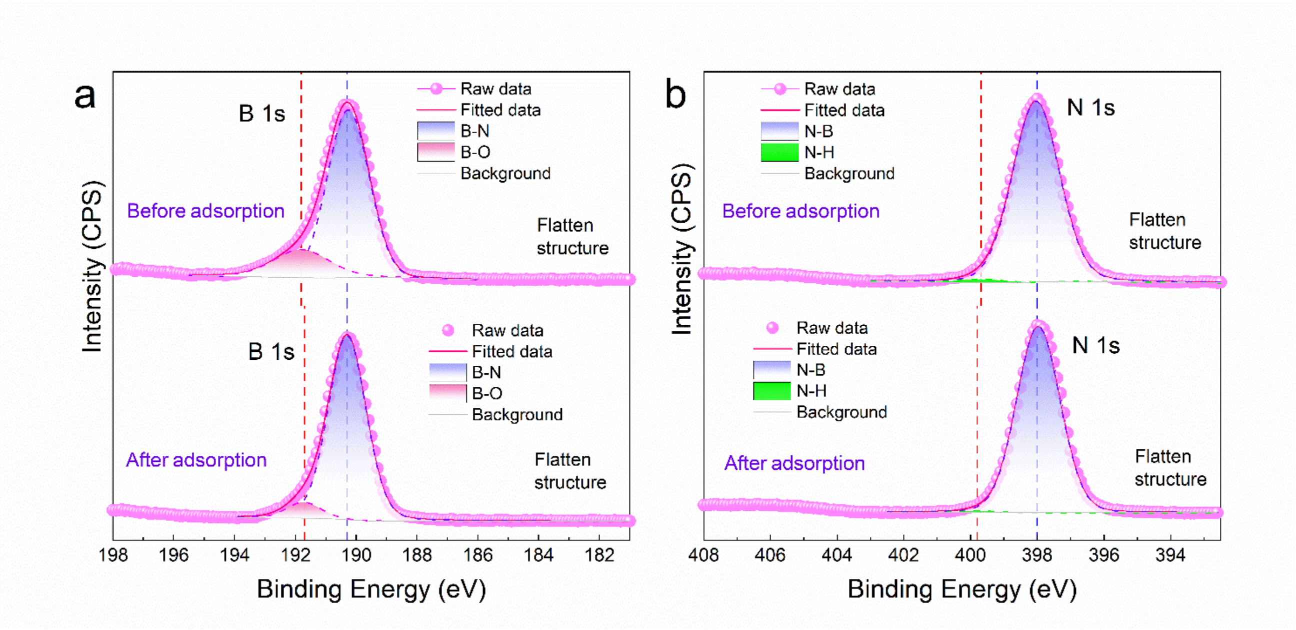

Figure S7 Comparison of XPS narrow scan spectra of BN aerogel with flatten microstructure before and after the adsorption of lead species, *Related to Figure 7.*

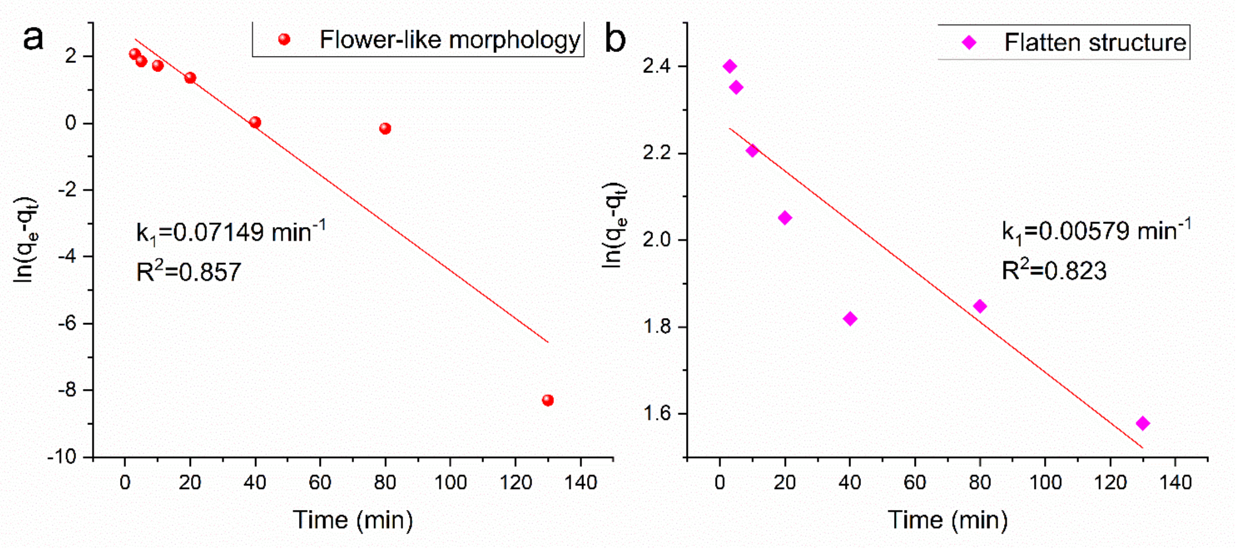

Figure S8 Pseudo-first-order fitting of the adsorption process in two cases. (a) corresponds to BN aerogel with flower-like morphology; (b) corresponds to BN aerogel with flatten structure, *Related to Figure 10.*

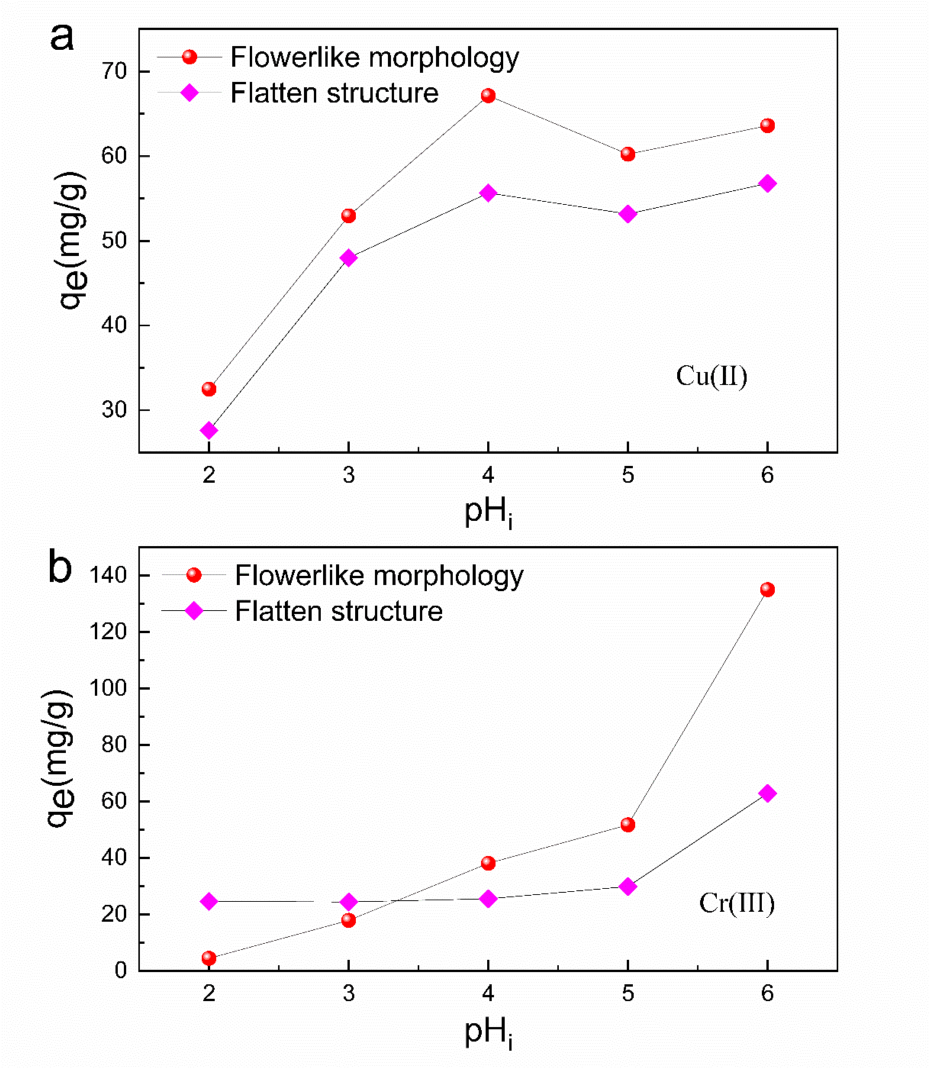

Figure S9 Influence of initial pH (pH_i) on the adsorption capacity of BN aerogels inheriting different structural hierarchy. (Initial concentration of Cu^{2+}/Cr^{3+}: 90 mg/L; volume of $CuCl_2$ /$CrCl_3$ solution: 30 ml; mass of adsorbent: 0.02 g.), *Related to Figure 6.*

Transparent Methods

Chemicals.

Boric acid (AR), melamine (CP), tert-butyl alcohol (AR), lead (Ⅱ) chloride (AR), copper (Ⅱ) chloride dihydrate (AR), chromium (Ⅲ) chloride hexahydrate (AR), sodium hydroxide (AR), hydrochloric acid (AR) and ethanol (AR) were procured from Sinopharm Chemical Reagent Co., Ltd. Acetoguanamine (98%) was purchased from Shanghai Macklin Biochemical Co., Ltd. All reagents in this work were used as received.

Preparation of BN aerogels.

In a typical synthesis, melamine (M, 0.608g), acetoguanamine (M*, 0.603g) and boric acid (B, 1.789g) were dissolved in 100ml solvent under 90℃(The molar ratio was fixed at M:M*:B=1:1:6). Two types of solvent were involved: one was pure water, and the other was the mixture of water and tert-butyl alcohol (TBA) with different volume ratios – the proportion of TBA varied from 10% to 30%. Herein, different solutions are marked by different symbols: I stands for solutions with pure water as solvent, and II represents solutions with the solvent being the mixture of water and TBA. In all cases, the obtained hot solutions were then cooled down under room temperature until white supramolecular gels formed. The as-formed wet gels were subsequently freeze-dried and converted to BN aerogels under 1400℃ for 4h with argon as shielding gas.

Tests of thermal conductivity.

Aerogel samples used for thermal conductivity are approximately 3 cm in diameter and 1cm in height. Thermal conductivity was tested via a transient hot-wire method at room temperature under atmospheric pressure.

Examination of wettability.

The wettability of aerogel was examined by measuring the contact angle. A contact angle tester (Dataphysics OCA20) was employed and the water droplet used for indication was 2μL.

Adsorption removal of heavy metal ions.

Initial stock solutions containing 1000 mg/L heavy metal ions were prepared by dissolving $PbCl_2$, $CuCl_2·2H_2O$ and $CrCl_3·6H_2O$ in distilled water in individual containers. These liquids were then diluted in different cases. In addition, 0.1M HCl and NaOH solutions were prepared for adjusting the pH value in next experiments.

To investigate the correlation between pH and adsorption performance, a series of heavy metal ions solutions (90mg/L) with their pH varying from 2 to 6 were prepared in the first step. Then 0.02 g BN aerogel was immersed in 30 ml of each solution. Given that two BN aerogel samples show different hydrophilicity, the above-mentioned heavy metal ions solutions were added equivalent amounts of ethanol (1ml) during their dilution processes from initial liquids, which ensures that both absorbents are lyophilic. The adsorption systems were left for 12 h before

the final concentration was measured, which ensures the equilibrium of final states. Throughout the adsorption process, the temperature was kept at 300K.

The adsorption capacity is calculated based on the following equation:

$$q_e = \frac{(C_o - C_e)V}{m}$$

C_o is the initial concentration of heavy metal ions; C_e is the equilibrium concentration after adsorption; V is the volume of solution; m is the mass of adsorbents; q_e is the equilibrium adsorption capacity.

The removal rate is measured according to the following equation:

$$Removal\ rate = \frac{C_o - C_e}{C_o} \times 100\%$$

C_o is the initial concentration of heavy metal ions; C_e is the equivalent concentration after adsorption.

The simulation of lead species distribution in $PbCl_2$ solution was run on Visual MINTEQ software.

To study the correlation between time and removal rate as well as residual concentration ratio, 0.2 g BN aerogel adsorbents were immersed in 50ml $PbCl_2$ solution (the initial concentration of Pb^{2+} is 100 mg/L). At 3min, 5min, 10min, 20min, 40min, 80min, 130min intervals, a tiny proportion of solution was quickly collected and filtered for concentration tests.

The fitting of adsorption kinetics is based on two models, which can be described as the following equations:

Pseudo-first-order model:

$$\ln(q_e - q_t) = lnq_e - k_1 t$$

Pseudo-second-order model:

$$\frac{t}{q_t} = \frac{1}{k_2\, q_e^2} + \frac{t}{q_e}$$

Where q_t is the real-time adsorption capacity; k_1 and k_2 are rate constants corresponding to two kinetic models.

For regeneration, two BN aerogels underwent different recovery treatment. In one case, two aerogel adsorbents were immersed in distilled water for three times and water was refreshed every 30 min; in the other case, they were immersed in hydrochloric acid (1mol/L HCl) for three times and the acid solution was refreshed every 30 min. Throughout the regeneration process, the temperature was kept at 40℃.

After regeneration, water and acid solution were poured out. The wet samples were freeze-dried and used in the following adsorption experiment.

Characterization of samples.

The microstructures of samples were characterized by using scanning electron microscope (SEM, Supra 35)

and transmission electron microscopy (TEM, Tecnai G^2 F20). X-ray diffraction was conducted on X-ray diffractometer (Rigaku D/max-2400). Nitrogen adsorption test was performed on Micromeritics ASAP 2020.

In the adsorption part, as the aerogel samples directly separated from Pb^{2+} solutions will inevitably have recrystallized lead species deposited within its inner structure, aerogel adsorbents were gently washed by alcohol before EDS and XPS tests, in order that the detected lead species are mainly adsorbed rather than deposited ones.

The concentration of heavy metal ions throughout this work was tested by inductively coupled plasma-optical emission spectroscopy (ICP-OES, Avio™ 200).
